# “Myc’ed Messages”: Myc Induces Transcription of E2F1 while Inhibiting Its Translation via a microRNA Polycistron

**DOI:** 10.1371/journal.pgen.0030146

**Published:** 2007-08-31

**Authors:** Hilary A Coller, Joshua J Forman, Aster Legesse-Miller

**Affiliations:** The Jackson Laboratory, United States of America

## Abstract

The recent revelation that there are small, noncoding RNAs that regulate the expression of many other genes has led to an exciting, emerging body of literature defining the biological role for these molecules within signaling networks. In a flurry of recent papers, a microRNA polycistron induced by the oncogenic transcription factor *c-myc* has been found to be involved in an unusually structured network of interactions. This network includes the seemingly paradoxical transcriptional induction and translational inhibition of the same molecule, the E2F1 transcription factor. This microRNA cluster has been implicated in inhibiting proliferation, as well as inhibiting apoptosis, and promoting angiogenesis. Consistent with its seemingly paradoxical functions, the region of the genome in which it is encoded is deleted in some tumors and overexpressed in others. We consider the possibility that members of this polycistronic microRNA cluster help cells to integrate signals from the environment and decide whether a signal should be interpreted as proliferative or apoptotic.

## Introduction

microRNAs are 21–23-nucleotide noncoding RNAs processed from double-stranded hairpin precursors present in a wide range of organisms including worms, plants, flies, and mammals [1,2]. microRNAs are loaded into the RNA-induced silencing complex and subsequently hybridize to complementary sequences in target mRNAs. This results in inhibition of mRNA translation or reduced message stability [3,4]. Microarray analyses suggest that individual microRNAs can regulate hundreds of genes [5]. This finding has raised the interesting possibility that microRNAs can coordinate complex cellular responses. One emerging model of the role of microRNAs is to maintain the robustness of genetic networks by ensuring that genes that ought to be “off” are downregulated not only via decreased transcription but also by translational inhibition (Text Box 1) [6,7]. Recently, however, a microRNA cluster was found to be involved in a complex network structured like a feed forward loop (described further in Text Box 2). This network appears to play a central role in controlling proliferation, apoptosis and tumorigenesis.

## The *miR-17–92* Cluster

Many microRNAs are present within the genome not as an individual microRNA but rather as clusters of multiple microRNAs [8,9]. Usually these clusters contain two or three genes but larger clusters exist, including the *miR-17–92* cluster, which contains seven mature microRNAs (*miR-17–5p, miR-17–3p, miR-18a, miR-19a, miR-20a, miR-19b,* and *miR-92–1*). These microRNAs are organized in a polycistron; that is, they are all transcribed as a single pri-microRNA, and then subsequently processed to form the individual microRNAs. The entire *miR-17–92* cluster is located within the third intron of an open reading frame termed the *C13orf25* (Chromosome 13 open reading frame 25) gene located at 13q31–q32. The close proximity of the six clustered microRNAs to each other (all six encompass only ∼800 base pairs of genomic DNA) makes it possible for all of the microRNAs to be transcribed and expressed similarly. In fact, a recent analysis of the expression of microRNAs in hematopoietic cell lines revealed similar expression patterns for all of the microRNAs in this cluster in the samples analyzed [8].

## Myc and E2F Induce Expression of *miR-17–92*, and Individual microRNAs Inhibit E2F Translation

**Figure 1:**
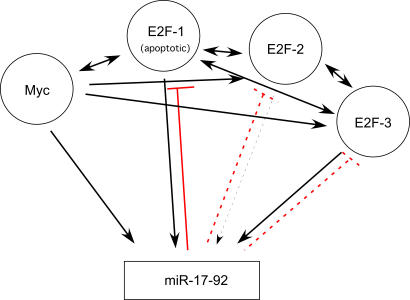
The Interactions among *c-myc*, the E2F1, E2F2, and E2F3 Transcription Factors and the microRNAs of the *miR-17–92* Complex Are Shown Black arrows indicate a transcriptional induction. Bidirectional arrows indicate mutual transcriptional induction. Darker arrows indicate stronger evidence of regulation while dashed arrows indicate that the evidence is less conclusive. Red lines indicate translational inhibition. Darker lines indicate stronger inhibition and dashed lines indicate weaker inhibition. Among members of the *miR-17–92* cluster, only *miR-17–5p* and *miR-20a* have been shown to inhibit E2F translation.

O'Donnell and colleagues demonstrated that activation of the oncogenic transcription factor *c-myc* induces the expression of microRNAs within the *miR-17–92* cluster [10]. Chromatin immunoprecipitation confirmed that *c-myc* binds to its recognition sites, E-boxes, upstream of this cluster. Serum stimulation of fibroblasts induced the expression of *c-myc* and the *miR-17–92* cluster with similar kinetics. Two of the microRNAs within the cluster, *mir17-5p* and *miR-20a,* downregulated the protein—but not mRNA—abundance of their predicted target, the transcription factor E2F1. Transfection of antisense oligonucleotides that inhibit *miR-17–5p* or *miR-20a* resulted in increased E2F1 protein levels without affecting E2F1 transcript abundance. Consistent with this finding, *c-myc* induction led to a strong increase in E2F1 transcript levels, but only a modest increase in E2F1 protein levels [10]. Since *c-myc* and E2F1 have been shown to activate each other's transcription [11–13], this established an unusually structured network in which *c-myc* activates the transcription of E2F1 while simultaneously inhibiting its translation (Figure 1). The importance of the *miR-17–5p/miR-20a* binding sites for E2F1 expression were demonstrated with reporter assays [10,14]. The 3′ UTRs of E2F2 and E2F3 were also regulated via *miR-17–5p/miR-20a* binding sites, but the downregulation of E2F1 protein levels was stronger than for E2F2 or E2F3 [14].

**Box 1. Genetic Buffering**

 Molecular networks that can withstand chance perturbations and reproducibly produce the same phenotypic results have been favored over the course of evolution [7]. Genetic buffering refers to the stabilization of molecular networks, making them less sensitive to chance fluctuations in the levels of specific molecules. The best-understood example of a specific molecule with the capacity to buffer a network is the chaperone protein *hsp90,* which can serve as a capacitor for the build-up of genetic variation. In *Drosophila,* when the heat-shock protein hsp90 is mutated or impaired, phenotypes are observed in nearly every adult structure [61]. Thus, widespread variation affecting *Drosophila* morphogenetic pathways exists but is usually silent because hsp90 buffers the variation. When hsp90 is incapacitated, for instance, under conditions of high temperature, cryptic variants may be revealed. This process may promote evolutionary change by increasing phenotypic variance under stressful conditions. Chromatin regulators have also been suggested to play a role as genetic buffers. A systematic screen for interaction pairs in C. elegans revealed that six genes involved in chromatin regulation interact with over one-quarter of all of the genes tested [62]. As in the case of hsp90, inactivation of these “hub” genes sensitized the animals to the phenotypic effects of knockdowns of many different genes.

 microRNAs have also been hypothesized to play a role in “canalization” or the increased robustness of phenotypic outcomes in the presence of noise. In some well-studied examples, microRNAs have been shown to reinforce the downregulation of transcripts in specific cell types or at times when the encoded proteins should not be present [6,7]. In several recent papers, microRNAs have been elegantly associated with regulatory loops that serve to reinforce lineage commitments, especially the irreversible commitment to a specific cell fate [63–65]. Under these conditions, a transient signal may result in one of two bistable states, characterized by either low microRNA levels and high target levels, or vice versa [64–66].

 In addition to reinforcing low expression of genes that are intended to be “off,” robustness can also be improved by minimizing “noise” in protein expression levels similar to hsp90’s effects at the protein level. The microRNAs of the *miR-17–92* complex have been proposed to help minimize noise in the levels of the E2F1 protein [7,23]. Because transcription is an inherently noisy process, frequent transcription coupled with infrequent translation results in lower intrinsic noise in protein levels compared with infrequent transcription [67–70]. Accordingly, in yeast, genes that are key regulators or essential have high rates of transcription and low rates of translation [71]. In this model, the *miR-17–92* cluster could limit the extent of translation, thus allowing the cell to make many mRNA copies but have a low and carefully controlled amount of protein. This model could be tested by determining whether *miR-17–92* actually affects inter-cell variability in E2F protein levels [72]. The amount of noise could be monitored with E2F1-YFP fusion proteins that include the relevant E2F 3′ UTR in the presence of scrambled 2′-O-methyl oligoribonucleotides, or 2′-O-methyl oligoribonucleotides that target individual microRNAs within the cluster.

To make matters even more complicated, the E2F transcription factors can also induce the microRNAs in the *miR-17–92* cluster [14,15]. Overexpression of E2F1 or E2F3 results in increased *miR-17–92* promoter activity [14,15]. Chromatin immunoprecipitation experiments by Woods and colleagues revealed stronger binding for E2F3 than E2F1 or E2F2 in three different regions of the *miR-17–92* cluster, confirming a likely role for E2F3 in *miR-17–92* expression. Because a control promoter at which all E2Fs are known to bind also resulted in stronger bands for E2F3 than for E2F1 or E2F2, this may reflect greater binding efficiency of the E2F3 antibody. Sylvestre and colleagues similarly concluded that immunoprecipitation followed by PCR resulted in bands of similar intensity for E2F1, E2F2, and E2F3 in one DNA region and a stronger band for E2F3 in a different region, which may also reflect higher binding affinity of E2F3 for this region, or higher binding affinity of the E2F3 antibody [14]. These findings suggest a complex network structure summarized in Figure 1.

## Can miR-17–92 Prevent Runaway myc-E2F Activation?

Because *c-myc* and E2F1, E2F2, and E2F3 can activate one another's transcription [11–13], one might imagine a cell in a runaway positive feedback loop in which *c-myc* induces the E2F transcription factors, which induce each other and, in turn, *c-myc*. This would result in excessively high levels of proliferative transcriptional regulators. High levels of *c-myc* have been clearly shown to have a tumor-promoting effect [16]. Just 2-fold differences in *c-myc* expression can affect cell size in flies or cell number in mice [17–20]. Indeed, dysregulated expression of *c-myc* is one of the most common abnormalities in human malignancy [21]. Thus, maintaining *c-myc* levels within a tight range can be considered critical for the prevention of cancer and accordingly, *c-myc* levels are tightly controlled at the level of transcription [16,22]. One hypothesized role for the *miR-17–92* cluster is to further the goal of carefully minimizing noise in *c-myc* levels. By ensuring that E2F1 protein levels do not rise precipitously in response to *c-myc* activation, *miR-17–92* may act as a brake on this possible positive feedback loop, thus helping to ensure tightly controlled expression of *c-myc* and E2F1 proteins [10,23]. From this perspective, the *miR-17–92* cluster might act as a tumor suppressor. Consistent with this hypothesis, loss of heterozygosity or deletion of the 13q31–32 region is observed in multiple types of tumors, including squamous cell carcinoma of the larynx [24], retinoblastoma [25], hepatocellular carcinoma [26], breast cancer [27], and nasopharyngeal carcinoma [28,29]. Copy number loss was observed frequently for the microRNAs of the *miR-17–92* cluster in breast cancers (21.9%), ovarian cancers (16.5%), and melanomas (20.0%) [30]. In addition, *miR-17–5p* in particular is expressed at low levels in multiple breast tumor cell lines and has antiproliferative effects on breast cancer cells [31].

**Box 2. Feed Forward Loops**

**Figure 2:**
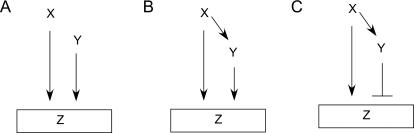
Two Molecules (X and Y) Can Both Regulate Molecule Z Independently (A) or Can Form a Feed Forward Loop (B) in Which X Regulates Z Both Directly and Indirectly via Y Feed forward loops can be coherent as shown in (B), in which case the direction of the regulation on Z is the same in either the direct or indirect path, or incoherent, illustrated in (C), in which case the two pathways have opposing effects on the target gene.

 Genetic networks contain repeated regulatory motifs including feed forward loops. In biological systems, this motif has been defined as two transcription factors, one of which regulates the other, and both of which regulate a third gene (Figure 2). These transcription factor-based loops can be “coherent,” in which case the sign of the direct path to the target gene is the same as the sign of the indirect path to the reporter. Alternatively, they can be “incoherent,” in which case the direct and indirect paths have opposing effects on the target. For transcription factor–based loops, both coherent and, less frequently, incoherent feed forward loops have been identified in S. cerevisiae and E. coli [73]. In human and mouse, incoherent loops involving microRNAs and their targets have been inferred to be common based on microarray data [23]. Both coherent and incoherent feed forward loops involving two transcription factors have been theoretically modeled and experimentally tested [73,74]. The two components contributing to expression of the reporter may be organized in an “AND” conformation, such that both are required, or an “OR” conformation, so that either is sufficient for expression. An incoherent feed forward loop can cause a pulse of reporter activity under certain conditions. In this situation, the upregulation kinetics are expected to be faster than if the two factors were acting independently [73,75]. Indeed, in experimental systems, it has been shown that an incoherent feed forward loop can lead to accelerated turn-on dynamics, even if embedded within other loops [74,75].

 We speculate that the structure of the feed forward loop involved in the *c-myc–miR-17–92*–E2F network may have an important impact on the kinetics of E2F1 activation. The network structure embedded within the *c-myc–miR-17–92*–E2F network may resemble a type I “incoherent” feed forward loop [73]. In the case of *miR-17–92,* microRNA-mediated inhibition of translation could allow a pulse of E2F activity. Once reaching a critical threshold, microRNAs could efficiently downregulate translation of the existing transcripts, thereby preventing a further rise in E2F1 abundance. This would allow a rapid shut-off of E2F1 activity that would be independent of the kinetics of E2F1 transcript degradation. Indeed, this mechanism might underlie the spike in E2F activity during cell-cycle entry [76]. Whether this network does in fact result in a rapid spike in E2F1 activity could be tested by a careful analysis of the time course of *miR-17–92* and E2F1 induction in response to *c-myc* activation. A finding that E2F1 levels and activity are rapidly induced and then decline following *c-myc* induction would support this model as a possible advantage of the network structure discovered.

## Individual Members of the *miR-17–92* Cluster Can Also Inhibit Apoptosis

Paradoxically, while the *miR-17–92* cluster has a putative role as an inhibitor of proliferation described above, the observed functional effect of overexpressing the microRNAs within the *miR-17–92* cluster is not to inhibit proliferation but rather to induce proliferation and/or inhibit apoptosis. As an example of the role of microRNAs in the *miR-17–92* cluster in inhibiting apoptosis, introduction of *miR-17–92* in conjunction with *c-myc* overexpression resulted in B-cell lymphomas characterized by an absence of the high levels of apoptosis normally associated with *c-myc*-induced lymphomas [32]. In in vitro studies, Sylvestre and colleagues demonstrated that overexpression of *miR-20a* decreased doxorubicin-induced cell death in a prostate cancer cell line, while inhibition of *miR-20a* with antisense oligonucleotides increased cell death after doxorubicin [14]. Matsubara and colleagues discovered that antisense oligonucleotides directed against *miR-17–5p, miR-20a,* or both result in increased apoptosis as measured by TUNEL assays in cancer cell lines overexpressing the *miR-17–92* cluster [33]. Little effect was observed when either *miR-17–5p* or *miR-20a* was introduced into cell lines that do not overexpress *miR-17–92,* or when antisense oligonucleotides against either *miR-18a* or *miR-19a* were introduced. These findings demonstrate that the microRNAs within this cluster have distinct functional effects, with at least *miR-17–5p* and *miR-20a* specifically inhibiting apoptosis.

Hayashita and colleagues also discovered that the microRNAs of the *miR-17–92* cluster promote proliferation. Transfection of the entire *miR-17–92* polycistron into lung cancer cells resulted in increased rates of cellular proliferation based on cell number. Introduction of the coding region of the *C13orf25* or the individual microRNAs *miR-18, miR-19a,* or *miR-20a* did not recapitulate the growth-promoting effects of the entire microRNA cluster [34]. Thus, one or more of the microRNAs in the cluster (but not *miR-18*, *miR-19a,* or *miR-20a*) may enhance proliferation via a mechanism distinct from the anti-apoptotic effect of *miR-17–5p* and *miR-20a*.

That the same microRNAs that inhibit E2F1 protein levels (*miR-17–5p* and *miR-20a*) also inhibit apoptosis suggests that E2F1 repression may be related to the inhibition of apoptosis conferred by the *miR-17–92* cluster. Indeed, the anti-apoptotic activity of this cluster has been proposed to reflect differences in the physiological effects of the different E2F transcription factors. E2F1, as opposed to E2F2 or E2F3, has been particularly associated with an apoptotic response. For instance, E2F1 is activated by the ATM/ATR DNA damage–signaling pathway [35]. And, E2F1-responsive sites are present in the promoters of caspases and other pro-apoptotic molecules [36,37]. Although E2F2 and E2F3 are also somewhat downregulated by the microRNAs in the cluster based on reporter assays [14], the effect on E2F1 is much stronger. Thus, the *miR-17–92* cluster has been proposed to inhibit apoptosis by decreasing E2F1 levels [14,15].

## Possible Role for Apoptosis Inhibition by *miR-17–92* in Spermatocytes

A physiological role for apoptosis inhibition mediated by *miR-17–92* has been suggested in spermatocytes [38]. In individual pachytene spermatocytes within a normal testis, high levels of E2F1 message but low levels of protein were associated on a cell-by-cell basis with high *miR-17–92* expression. This has been suggested to reflect the particular needs of spermatocytes, cells in which meiotic recombination induces extensive crossing over of sister chromatids and multiple double-strand breaks, which would be expected to result in apoptosis [38]. Inhibition of E2F1 by *miR-17–92* could be important for preventing apoptosis in these cells during meiotic recombination. These findings suggest that the *miR-17–92* cluster may be important for inhibiting apoptosis under conditions in which it would be detrimental to the organism. In addition, this same mechanism for apoptosis inhibition may be associated with tumorigenesis, as described further below.

## *c-myc* Induction of *miR-17–92* Induces Tumor Angiogenesis

*c-myc* has been reported to promote neovascularization via upregulation of pro-angiogenic VEGF [39] and downregulation of anti-angiogenic thrombospondin-1 (tsp-1) [40,41]. In addition, Dews and colleagues recently discovered that the *miR-17–92* microRNA cluster also plays a role in *c-myc*–induced angiogenesis in a *ras–myc* tumorigenesis model. When engrafted into mice, cells overexpressing *ras* form small tumors while overexpression of both *c-myc* and *ras* results in larger tumors with much more robust neovascularization [42]. In cell culture, *miR-17–92* levels were elevated in the presence of overexpressed *c-myc,* and levels of predicted targets tsp-1 and connective tissue growth factor (CTGF) declined. Transfection of antisense oligoribonucleotides revealed that *miR-18* is especially important for CTGF regulation and *miR-19* is important for tsp-1 regulation. Inhibition of *miR-17, miR-20a,* or *miR-92* had no effect on the levels of angiogenesis target genes. Further, cells overexpressing both *ras* and *miR-17–92* had lower levels of CTGF and formed tumors that were larger and more vascular than tumors formed by cells expressing only *ras*.

## *miR-17–92* and *c-myc* Are Overexpressed in the Same Tumors

While deletion of 13q31–32 has been observed in some tumors as described above [24–29], paradoxically, amplification at 13q31–32 is also frequently observed in multiple tumor types, including liposarcoma [43], diffuse large B-cell lymphomas [44], and colon carcinomas [45]. Fine mapping of the 13q31–32 region in diffuse large B-cell lymphomas revealed that *C13orf25* is elevated in lymphoma cell lines and patients [32,46]. Overexpression of *miR-17–92* may be particularly tumor-promoting when *c-myc* is also activated. Amplification of *miR-17–92* in the B-cell lymphoma tumor type is consistent with a role for *miR-17–92* in conjunction with *c-myc,* because human B-cell lymphomas are often characterized by high *c-myc* expression [47]. Elevated expression levels of *miR-17–92* members has also been associated with lung cell tumors, especially those with *c-myc* amplification. *miR-17–92* overexpression was detected at the RNA level and in some cases at the DNA level as well, suggesting amplification events [34]. In yet another study, a human mantle cell lymphoma was shown to contain genomic amplification of both *c-myc* and *miR-17–92* [48].

One simple explanation for the concordance between high levels of *c-myc* and *miR-17–92* would be that *c-myc* directly induces *miR-17–92*. But this is unlikely to represent the entire story, as genomic amplification of the *miR-17–92* cluster has also been observed [34,48]. In addition, introduction of *miR-17–92* hastens lymphomagenesis in Eμ-myc overexpressing mice [32]. As described above, lymphomas with high levels of *miR-17–92* exhibited less apoptosis than typically found in *c-myc*–overexpressing tumors, consistent with the model that *miR-17–92* overexpression inhibits apoptosis. This finding demonstrates that *miR-17–92* overexpression confers a further selective advantage in a high *c-myc* background.

## *miR-17–92* as an Integrator of Proliferative versus Apoptotic Signals?

We conclude by hypothesizing that the unusual structure of the *c-myc*–*miR-17–92*–E2F network may help the cell to integrate external signals to make a cell fate decision. *c-myc* activation can result in proliferation, and indeed, *c-myc* levels are often markedly elevated in tumors. However, in different cellular contexts, *c-myc* can be a potent inducer of apoptosis [49–55]. In a mouse model with inducible *c-myc,* activation of *c-myc* in pancreatic β cells induced uniform β cell proliferation, but also overwhelming apoptosis, thus counteracting the oncogenic potential of *c-myc* [56]. When *c-myc* was activated in conjunction with overexpression of *Bcl-x_L_,* which suppresses *c-myc*–induced apoptosis, then *c-myc* triggered rapid, uniform, and reversible progression to tumorigenesis. *c-myc/Bcl-x_L_*-induced β cell tumors also contained an extensive network of blood vessels that regressed after *c-myc* was switched off.

We speculate that the *miR-17–92* cluster may also have an important role in the balance between a proliferative versus apoptotic response to *c-myc* induction. Under normal proliferative conditions, induction of *miR-17–92* in response to *c-myc* activation could serve as a brake on excessive proliferation. *miR-17–92*–mediated E2F1 inhibition would attenuate the hypothetical positive feedback loop of *c-myc*–E2F activity. The absence of the *miR-17–92*–mediated dampening of proliferative signals could be tumorigenic, and this might explain the deletions in the 13q31–32 region in multiple types of tumors [24,25,28]. *miR-17–92* induction would also limit apoptosis under normal proliferative conditions.

Under other conditions, for instance, in stressful situations, we hypothesize that *c-myc* activation might occur in the absence of *miR-17–92* induction. Our model would predict that *miR-17–92* would not be induced, or would be induced less robustly, under conditions in which *c-myc* activation results in apoptosis. The molecular mechanisms that control *miR-17–92* in response to *c-myc* induction are thus of particular interest. One possible model would be that both *c-myc* and E2F are required to induce expression of *miR-17–92,* and that the presence of both transcription factors signals to the cell that *c-myc* activation should be interpreted as a proliferative rather than apoptotic signal. In this model, E2F transcription factors would act as sensors for whether conditions are suitable for proliferation [14,15]. Monitoring both *miR-17–92* expression and the occupancy of the *miR-17–92* promoter by *c-myc* and E2Fs in response to proliferative and apopotic signals would likely shed light on whether the expression of this cluster reflects an “AND” signaling switch.

We further hypothesize that *miR-17–92* has a distinct effect in a *c-myc*–overexpressing environment. *c-myc* activation induces proliferation that is simultaneously held in check by apoptosis. If a cell has constitutive *c-myc* overexpression, then this would override the anti-proliferative effects of the *miR-17–92* cluster. If the same cell also acquires constitutively high levels of *miR-17–92,* it will lose the apoptotic response that keeps its proliferative capacity in check, and may proceed to proliferate and form a tumor. Thus, overexpression of both *c-myc* and *miR-17–92* would be expected to create a tumor-promoting environment. Indeed, overexpression of both *c-myc* and *miR-17–92* has been observed in the same tumors [34,48], as described above. Under these conditions, the pro-angiogenic effects of *c-myc* and *miR-17–92* may also cooperate with the anti-apoptotic effects of the *miR-17–92* cluster to simultaneously create multiple conditions conducive for a tumor growth.

## Future Research

In summary, a series of elegant recent papers has illuminated a fascinating and unexpected network of interactions involving the *c-myc* and E2F transcription factors, and the members of a microRNA cluster. This network may be organized in the format of an incoherent feed forward loop, in that *c-myc* induces E2F1 transcription while repressing E2F1 translation. The microRNAs within this cluster may act as a brake on proliferation, inhibiting apoptosis and promoting angiogenesis. We look forward to further research that will clarify the roles of *c-myc,* different E2F transcription factors, and other regulators in controlling expression of the *miR-17–92* cluster. In particular, experiments addressing whether *c-myc* and the E2Fs act synergistically or independently to control *miR-17–92* expression would help to define its potential role as a signal integrator. It will also be interesting to determine whether *miR-17–92* plays a role in tumorigenesis mediated by other genetic mechanisms. For instance, in chronic myeloid leukemias, expression of the *miR-17–92* cluster was downregulated by RNAi directed against the pro-oncogenic fusion protein bcr-abl [57]. Other microRNAs have been implicated in tumorigenesis, either as oncogenes or tumor suppressors [58–60]. The mechanisms by which these microRNAs affect tumorigenesis, in particular, whether they affect the same or different molecules and employ the same or different molecular circuitry, would shed light on the key elements in the transition to tumorigenesis. Recent analysis of gene expression patterns between microRNAs and their targets suggest that networks of this type, in which the expression of the microRNA and its targets are positively correlated, are common in human and mouse, especially in neural tissues [23]. This makes it of particular importance to discover the potential advantages conferred by this seemingly paradoxical network structure.
